# An investigation of lower urinary tract symptoms and its potential associated factors in adult Chinese women with zero-inflated negative binomial regression model

**DOI:** 10.1097/MD.0000000000017409

**Published:** 2019-10-04

**Authors:** Tao Xu, Lei Zhang, Zhiyi Li, Lan Zhu, Shaomei Han

**Affiliations:** a Department of Epidemiology and Statistics, Institute of Basic Medical Sciences, Chinese Academy of Medical Sciences & School of Basic Medicine, Peking Union Medical College; b Department of Gynecology and Obstetrics, Peking Union Medical College Hospital, Peking Union Medical College; c Department of Gynecology and Obstetrics, Peking University First Hospital, Beijing, People's Republic of China.

**Keywords:** bother, China, lower urinary tract symptoms, potential associated factors, zero-inflated negative binomial model

## Abstract

Lower urinary tract symptoms (LUTS) have detrimental impact on health-related quality of life. This study has 2 aims: first to identify the optimum model for LUTS study and then to explore the potential associated factors of LUTS and bother LUTS with the optimum model among adult women in China.

The survey was conducted in 6 regions of China between February and July 2006. A modified Chinese Bristol Female LUTS questionnaire was administered. The number of LUTS was the main outcome measure. The fitting goodness was compared to identify the optimum model with likelihood ratio test statistics. Zero-inflated negative binomial (ZINB) model was used to explore the potential associated factors of LUTS and bother LUTS.

Of all 18,992 respondents, 55.5% of respondents reported one (any LUTS) or more LUTS (mixed LUTS) and 36.5% of respondents reported one or more bother LUTS. With the largest log likelihood and smallest AIC and BIC, ZINB model showed the best goodness of fit. In the ZINB model, we identified multiple associated factors for any LUTS and mixed LUTS; older age (β≥0.2), overweight [β = 0.059, 95%CI (0.016∼0.102)], obese [β = 0.143, 95%CI (0.087∼0.198)], postmenopausal status [β = 0.099, 95%CI (0.023∼0.175)], prolonged labor [β = 0.188, 95%CI (0.104∼0.272)], constipation [β = 0.309, 95%CI (0.262∼0.357)], coexisting pelvic organ prolapse (POP) [β = 0.348, 95%CI (0.224∼0.473)], diabetes (β = 0.178, 95%CI (0.100∼0.257), hypertension [β = 0.092, 95%CI (0.041∼0.143)], smoking (β = 0.192, 95%CI (0.127∼0.258) and alcohol consumption [β = 0.063, 95%CI (0.001∼0.126)] increased the odds of mixed LUTS. We identified multiple associated factors for bother LUTS and mixed LUTS; older age (β ≥ 0.1), prolonged labor [β = 0.153, 95%CI (0.031∼0.275)], constipation [β = 0.359, 95%CI (0.292∼0.426)] coexisting POP (β = 0.212, [95%CI (0.031∼0.393)], diabetes [β = 0.154, 95%CI (0.030∼0.278)], and smoking [β = 0.169, 95%CI (0.076∼0.262)] increased the odds of bother mixed LUTS.

ZINB model was the optimum model to explore the potential associated factors of LUTS. Older age, coexisting POP and constipation were both closely related to any and bother LUTS, also the severity of LUTS. Compared to nulliparity, single or multiple deliveries and women who had perineal laceration had nothing to do with the severity of LUTS.

## 1 Introduction

Lower urinary tract symptoms (LUTS) have been reported because of high prevalence and detrimental impact on health-related quality of life.^[1]^ It is said that one-fifth of adult women may be bothered by moderate to severe LUTS, and one-tenth of adult women have newly developed LUTS, often along with resulting substantial bother and interference with daily activities.^[2,3]^ A previous study had ever showed that the prevalence of any LUTS was 55.5%, among which 14.6% to 29.9% of respondents reported a moderate to severe impact on their life quality and 4.2% to 11.5% of them reported severe bother in Chinese women, which was the largest epidemiology study up to date.^[4]^ The pathogenesis of LUTS is considered a multi-factorial process.^[5]^ Assessing a more detailed evaluation of the potential risk factor have key clinical relevance and prevention significance. If a subject has suffered from one or more LUTS symptom items, she is often considered as being in LUTS status. Accordingly, all respondents were dichotomized as 2 subsets: not in LUTS status and in LUTS status. And then all respondents in LUTS status were analyzed with same method regardless of the number of LUTS symptoms. However, as LUTS contained many kinds of symptoms, these traditional analytical methods could not utilize all information regarding the heterogeneous LUTS symptoms among each subject and then would result in inability to assess the severity of LUTS status. Actually, the number of LUTS symptoms can utilize all information to assess the severity of LUTS status. Considering the data type, the number of LUTS symptoms is a type of count data, in which the observations can take only the non-negative integer values {0, 1, 2, 3 ...}. Four count data models: Poisson model, negative binomial (NB) model, Zero-inflated Poisson (ZIP) model and Zero-inflated negative binomial (ZINB) model could be used to model the outcome measures. But they have different application circumstances. This study has 2 aims: first to compare the fitting goodness of 4 count outcomes models to identify the optimum model for LUTS study and then to explore the potential associated factors of LUTS and bother LUTS with the optimum model among adult women in China.

## 2 Methods

### 2.1 Study design and respondents

The data were from a national population-based survey sample on urinary incontinence (UI) in 18,992 adult women in China. This cross-sectional survey was conducted between February and July 2006. The survey's main objective was to explore the nationwide prevalence, subtypes, associated risk factors and distribution of UI and LUTS among adult Chinese adult women aged above 20 years old across different Chinese areas, and aimed to evaluate the epidemiology of UI and LUTS in adult Chinese women. The longer goal was to provide evidence for constructing Chinese future national medical preventive strategies and treatment for LUTS. The population-based sample was selected by a 2-phased process. Phase 1 as a pilot survey assessed a representative sample of approximately 5221 respondents who were interviewed face to face by trained interviewers. The eligibility criteria included that women had to be ages of 20 years or older and had lived in the registered areas for at least 10 years. We excluded those who were diagnosed with cognitive impairments. The sampling process of Phase 2 was multi-staged and stratified according to geographic region (northeast, northwest, north, east, southwest, and central-south China), degree of urbanization and economic conditions. The study was approved by the review board of Peking Union Medical College Hospital. Written informed consent was obtained from each participant before data collection. All methods of the study were conducted in accordance with the *Helsinki Declaration* and relevant guidelines and regulations.

### 2.2 Assessment of LUTS

The diagnostic criteria in this study are consistent with the standards recommended by the 2002 International Continence Society (ICS) guidelines.^[6]^ We used a modified Bristol Female Lower Urinary Tract Symptoms questionnaire that consisted of 2 parts.^[7]^ Part 1 included general information and part 2 was an adapted Chinese version of the International Consultation on Incontinence Questionnaire-Female Lower Urinary Tract Symptoms (ICIQ-FLUTS). This questionnaire included an assessment of 10 kinds of LUTS: nocturia, daytime frequency, urgency, urgency urinary incontinence (UUI), stress urinary incontinence (SUI), other incontinence, pain or burning, hesitancy, straining, and intermittency. Each participant was asked to rate how often she experienced LUTS during the previous 4 weeks and, if LUTS were experienced, to what degree the symptoms bother. All women reporting LUTS were asked to describe the frequency of the symptoms. Nocturia is the complaint that the person has to awaken at night one or more times to void and was defined as 2 or more micturitions per night in our study. Daytime frequency is the complaint by the patient that she voids too often by day. Other incontinence was defined as UI without symptoms of UUI or SUI. The bother associated with each symptom was evaluated using a scale ranging from 0 (not bothered at all) to 10 (greatly bothered). Standardized training before interviewing and regular quality control checks during and after interviewing were conducted.

### 2.3 Statistical analysis

All analyses were performed using SAS 9.2 (SAS Institute, Cary, NC). *P* values less than .05 were considered statistically significant. The number of any LUTS and the number of bother LUTS were the outcome measures. Four count data models were conducted, including Poisson regression model, NB regression model, ZIP model and ZINB model.

All 4 count data models could be used to model the outcome measures. But they had different application circumstances. In statistics, both Poisson regression and NB regression were generalized linear model forms of regression analysis used to model count data. Poisson model assumed the response variable Y follows a Poisson distribution. NB model assumed the response variable Y follows a NB distribution and could handle over dispersion of the count outcome. However, in the circumstances that there were excessive zero counts, the fitting goodness of Poisson model and NB model would be greatly compromised and the estimation of parameters of two models might be biased. Neither Poisson model nor NB model could handle excessive zero counts, which could be solved in ZIP model and ZINB model. Especially, ZINB model could solve both over dispersion and excessive zero counts at the same time. Both ZIP model and ZINB model were two-part models, consisting of logit section and count model section in order to account for excess zero counts.^[8]^ ZIP model supposed that: 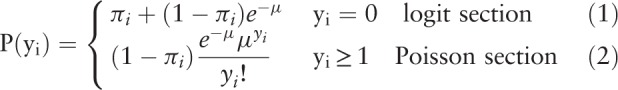


At the same time, the probability density function of ZINB model was that: 
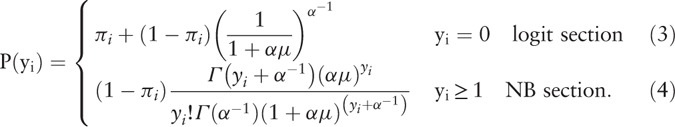


In Eq. (1) to equation (4), ln and logit link functions were used for parameters *μ* and *π*_*i*_. ln(μ) = B_i_β. logit(π_i_) = ln[(π_i_/1-π_i_)] = G_i_γ.

A covariate might have different impact on the prevalence and severity of LUTS in 2 sections of ZI models. The logit section in ZI models explored whether potential determinants influenced the prevalence rate of LUTS or bother LUTS. In the logit section, the explanations of regression coefficients were similar to those in multivariate logistic regression. Odds Ratios (ORs) were used to explain the relational degree between potential determinants and the prevalence rate of LUTS. The larger OR means larger strength of the relationship. And then the Poisson or NB section assessed the influence of potential determinants on the severity of LUTS or bother LUTS. In the Poisson or NB section, the explanations of regression coefficients were same as those in the traditional Poisson or NB regression models. Betas were used to explain the relational degree between potential determinants and the severity of LUTS. The larger absolute value of beta means larger strength of the relationship.

Over dispersion and excessive zero counts were not the necessary attributes for all count outcome measures. So, it was not sure about whether the number of LUTS symptoms and the number of bother LUTS were over dispersed or with excessive zero counts before doing the appropriate tests. The *O* test, Vuong test and likelihood ratio test statistics were used to explore whether the outcome measure was over dispersed or with excessive zero count, and to compare the fitting goodness of four count data models and finally determine the optimum model in this study.

The *O* test was used to identify whether the number of LUTS is over-dispersed.^[9]^ The *O* test statistic was calculated with the equation (5): 
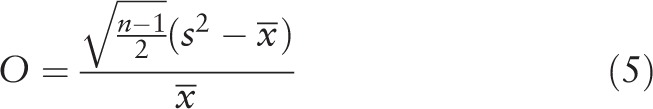


In equation (5), *n* was the sample size, 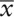
was the mean of the count measure, and *s*^*2*^ was the variance of the count measure. When *O* equal to or was above than 1.96, the outcome measure was over dispersed and then the Poisson model or ZIP model was not fit for the outcome measure.

The Vuong test was conducted to compare non-nested models, NB and ZINB models, in order to evaluate whether there were excess zero count.^[10,11]^ The Vuong test statistic was calculated with the equation (6): 
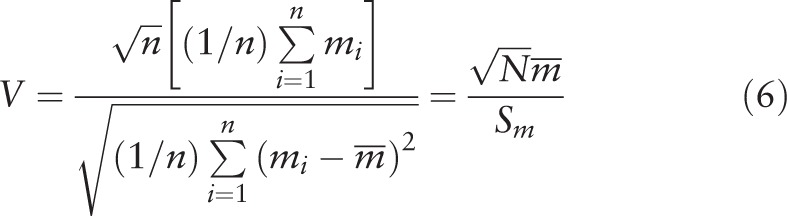


In equation (6), *m*_*i*_ *=* log[*f*1(*y*_*i*_)/*f*2(*y*_*i*_)], 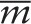 was the mean of *m*_*i*_, *S*_*m*_ was the standard deviation of *m*_*i*_. *f*1 and *f*2 were the probability density function of Poisson model and NB model. When *V* equal to or was above than 1.96, the outcome measure has excessive zero counts and the NB model was not fit for the outcome measure.

The fitting goodness of regression models were further evaluated by likelihood ratio test statistics: log-likelihood, Akaike's Information Criterion (AIC) and Bayesian information criterion (BIC).^[12]^ Log-likelihood was the test statistic of the likelihood ratio test and the larger log likelihood meant better fitting goodness of models. AIC was calculated with the equation (7): 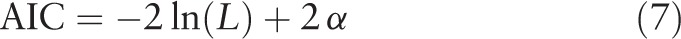


BIC was calculated with the equation (8): 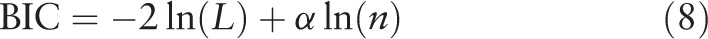


In Eq. (7) and equation (8), *L* was the likelihood function, ln(*L*) was the log likelihood, *α* was the number of estimable free parameters, *n* was the sample size. The smaller AIC and smaller BIC meant better fitting goodness of models.

## 3 Results

A total of 18,992 respondents (94.96%) were included who aged from 20 to 99 years and the mean age of 44.9 ± 15.9 years. We analyzed the age distribution between the total adult women of the 2006 National Census and this study population which shows a good population sampling ration (Table 1). Table 2 shows the general information of the respondents.

**Table 1:**
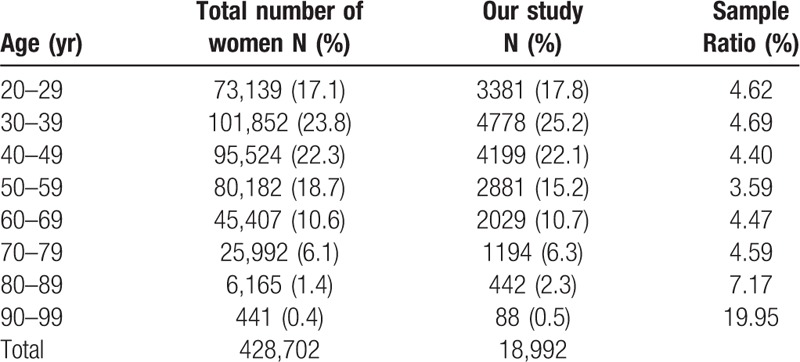
The age distribution of the 18,992 subjects interviewed.

**Table 2:**
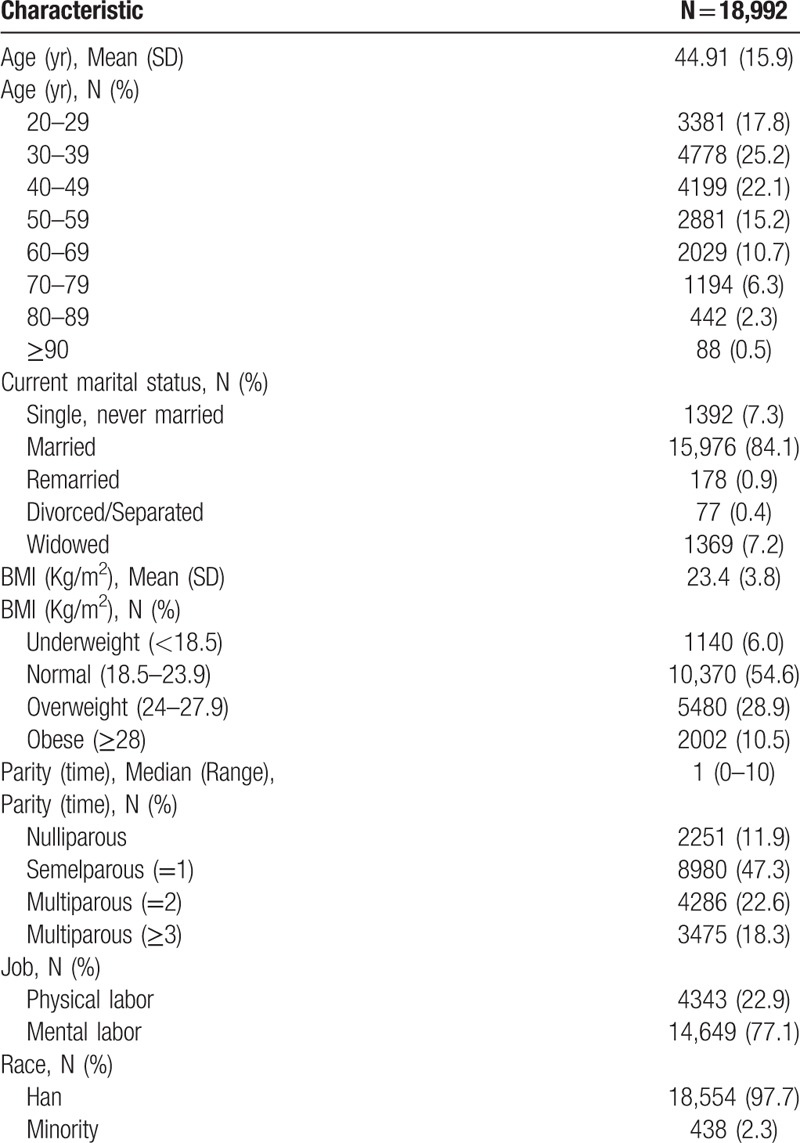
Characteristics of the 18,992 subjects interviewed.

### 3.1 Potential associated factors of any and mixed lower urinary tract symptoms

Of all 18, 992 respondents, 44.5% did not report any LUTS. 55.5% of respondents reported one (Any LUTS) or more LUTS (Mixed LUTS). The larger number of symptoms means the lower proportion of respondents. The largest number of LUTS was 8. The mean number of LUTS was 1.16 ± 1.46 and the variance (2.13) was significantly bigger than the mean. The over-dispersion test statistic *O* was 80.710 and *P* value was less than .001, which showed that the number of LUTS was over-dispersed. And both Poisson distribution and ZIP distribution were worse than corresponding NB distribution and ZINB distribution for fitting this count outcome, the number of LUTS. The Vuong test statistic *Z* was 32.65 and *P* value was less than .001, which showed that there were too many zero counts to be accounted for with traditional NB model. Table 3 showed that the fitting goodness statistics of four count outcomes models. With the largest log likelihood, smallest AIC and smallest BIC, ZINB model showed the best goodness of fit. ZINB model was the best model to fit the number of LUTS.

**Table 3:**
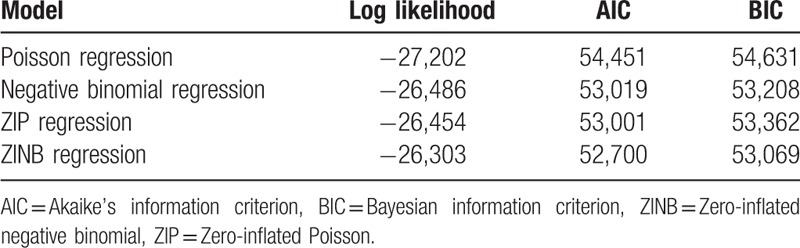
The fitting goodness statistics of regression models of lower urinary tract symptoms.

Associated factors of LUTS according to ZINB analysis are shown in Table 4. It was clear in the logit section that older age, overweight, obese, postmenopausal status, perineal laceration, constipation, alcohol consumption increased the odds of any LUTS and nulliparity cut down the odds of any LUTS. In the negative binomial section, we identified multiple associated factors for any LUTS and mixed LUTS (the severity of LUTS); older age, overweight, obese, postmenopausal status, prolonged labor, constipation, coexisting pelvic organ prolapse (POP), diabetes, hypertension, smoking and alcohol consumption increased the odds of mixed LUTS. Older age, constipation and coexisting POP were both strong predictors (β ≥ 0.3). Our study showed a higher risk of mixed LUTS in women with prolonged labor. However, in comparison with spontaneous vaginal single births, we did not find any difference between women who had single or multiple deliveries and women who had perineal laceration.

**Table 4:**
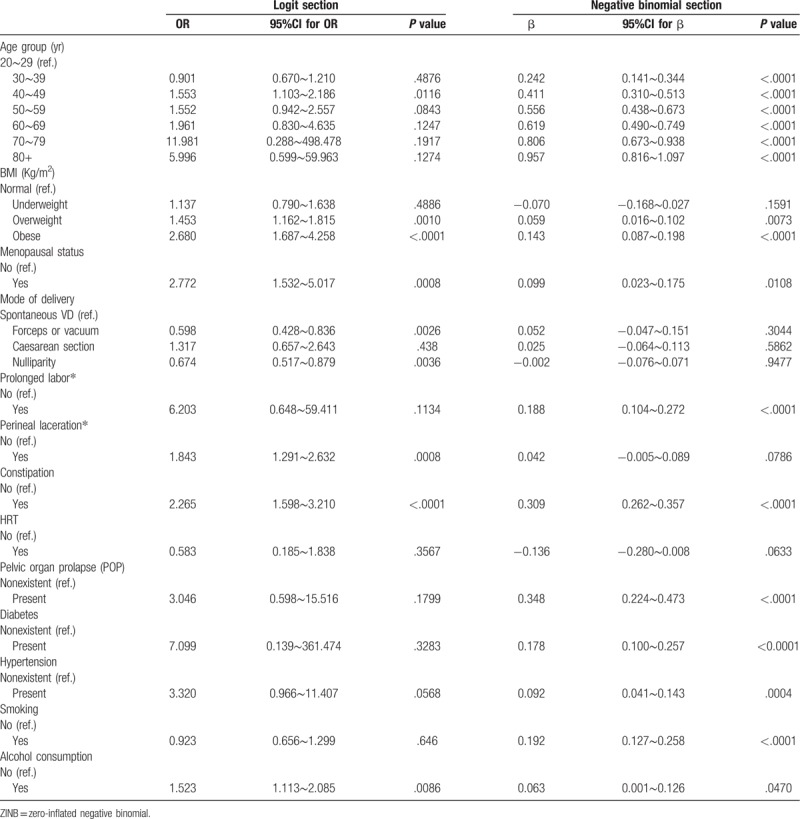
Potential associated factors of lower urinary tract symptoms (ZINB Model).

### 3.2 Potential associated factors of any and mixed bother lower urinary tract symptoms

Of all 18,992 respondents, 63.5% did not report any bother LUTS. 36.5% of respondents reported one or more bother LUTS. The larger number of symptoms means the lower proportion of respondents. The largest number of bother LUTS symptoms was 8. The mean number of bother LUTS was 0.89 ± 1.58 and the variance (2.50) was significantly bigger than the mean. The over-dispersion test statistic *O* was 175.882 and *P* value was less than .001, which showed that the number of bother LUTS was over-dispersed. Both Poisson distribution and ZIP distribution were worse than corresponding NB distribution and ZINB distribution for fitting this count outcome, the number of bother LUTS. The Vuong test statistic *Z* was 45.16 and *P* value was less than .001, which showed that there were too many zero counts to be accounted for with traditional NB distribution. Table 5 showed that the fitting goodness statistics of four count outcomes models. With the largest log likelihood, smallest AIC and smallest BIC, ZINB model showed the best goodness of fit. ZINB model was the best model to fit the number of bothers LUTS.

**Table 5:**
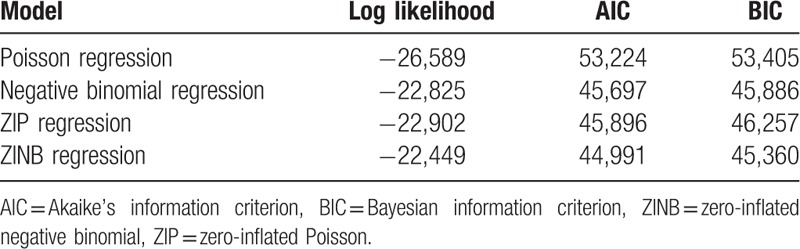
The fitting goodness statistics of regression models of bother lower urinary tract symptoms.

Associated factors of bother LUTS according to ZINB analysis are shown in Table 6. It was clear in the logit section that older age, overweight, obese, postmenopausal status, prolonged labor, perineal laceration, constipation, coexisting POP, hypertension, alcohol consumption increased the odds of bother LUTS, nulliparity (OR = 0.792, 95%CI: 0.665∼0.943) cut down the odds of bother LUTS. In the negative binomial section, we identified multiple associated factors for bother LUTS and mixed LUTS (the severity of LUTS); older age (β ≥ 0.1), prolonged labor, constipation, coexisting POP, diabetes, and smoking increased the odds of bother mixed LUTS, and underweight reduced the odds of bother mixed LUTS. Older age and constipation were both strong predictors (β ≥ 0.3). Our study showed a higher risk of mixed bother LUTS in women with prolonged labor. However, in comparison with spontaneous vaginal single births, we did not find any difference between women who had single or multiple deliveries and women who had perineal laceration.

**Table 6:**
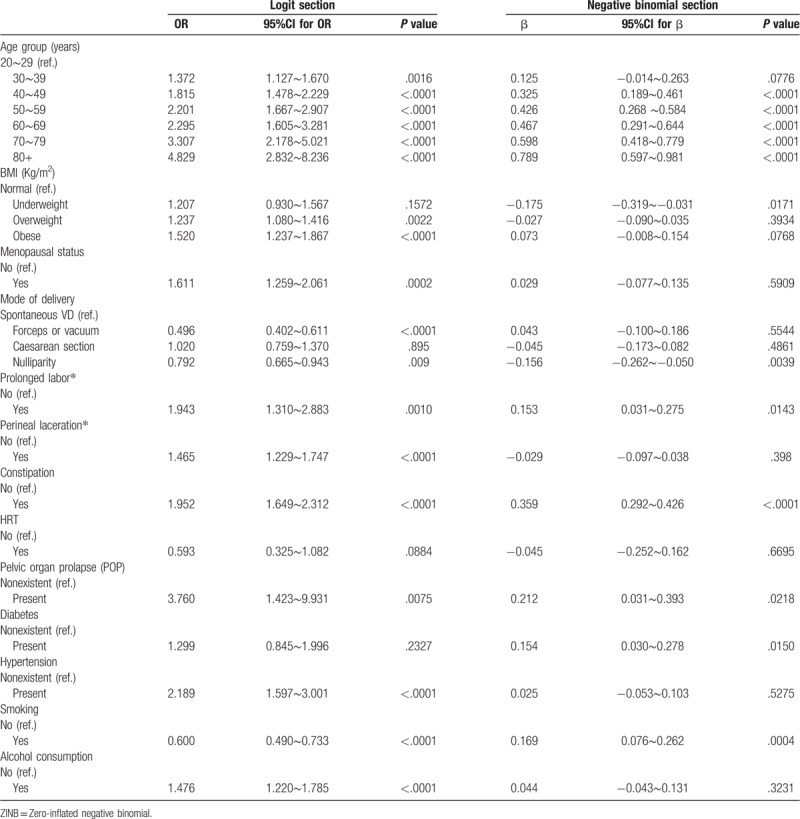
Potential associated factors of bother lower urinary tract symptoms (ZINB Model).

## 4 Discussion

To our knowledge, this is the first study to explore the application of ZI models in LUTS based on a large-scale population survey and examined the influence of each determinant on whether LUTS or not and the severity of LUTS status. *O* test, Vuong test, fitting goodness statistic and likelihood ratio test indicated that ZINB model was the best model for the number of LUTS with proximately half or even two-thirds zero counts. ZINB model could explore the influence of potential determinants on both the prevalence of LUTS and the severity of LUTS at the same time.

Many studies of LUTS has reported that the potential associated factors may be age, race, micturition habits, lifestyle factors, and may other pathologic conditions.^[5]^ We reported different associated factors using the ZINB model, prolonged labor, POP, diabetes increased the odds of mixed LUTS or the severity of LUTS, however, not related to the onset of any LUTS. This suggests that pelvic floor function in women with the history of prolonged labor and POP may not easily recover and experience LUTS progression or mixed LUTS, Which should be targeted for medical intervention. Prolonged labor may cause repeated injury to the muscles, nerves, and connective tissue of the pelvic floor and have a negative impact on bladder control. Having either incontinence or prolapse may be associated with an increased risk of developing the other condition.^[5]^ Perhaps women with advanced POP experienced more obstructive urinary symptoms, as reported by Rosenzweig et al and Raz et al.^[13,14]^ Family studies and twin studies demonstrate that LUTS and POP are both heritable.^[5]^ Animal studies have shown that long term increased serum glucose induces neuronal apoptosis that favors parasympathetic neuron loss compared to sympathetic.^[15]^ Such an unbalanced loss of autonomic neurons might induce an oversupply of sympathetic tone compared to parasympathic efferent activity resulting in increased bladder neck obstruction and reduced bladder power, which combined might produce an increase in obstructive symptoms as noted. An emerging consensus of investigators suggests that diabetic linked bladder neuropathy was principally a sensory defect resulting in a delayed desire to void due to the absence of urgency.^[16]^

The relationship between alcohol consumption and LUTS has been evaluated in a number of cross sectional studies but the findings were inconsistent. Alcohol consumption has been shown to be inversely related to severity of LUTS in some studies while others demonstrated a positive relationship between heavy alcohol consumption and LUTS.^[17–19]^ Our study also showed that alcohol consumption was related to the onset of bother LUTS, however inversely related to severity of LUTS. Diabetes was also related to the mixed bother LUTS because it may be related to the onset of storage and voiding function.

Due to the cross-sectional design, the associated factors observed in the present study could not be considered as causes of LUTS. Some of the results should be interpreted cautiously. Despite this limitation, all fitting goodness test statistics produced same findings that ZINB model was the optimum model to explore the potential associated factors of LUTS. Older age, coexisting POP and constipation were both closely related to any and bother LUTS, also the severity of LUTS. Compared to nulliparity, single or multiple deliveries and women who had perineal laceration had nothing to do with the severity of LUTS, which more studies were needed to reveal the reason. Understanding epidemiology of LUTS in adult Chinese women was the first step and might help direct treatment resources and provide preventive steps. The study results provided evidence for constructing Chinese future national medical preventive strategies and treatment for LUTS.

## Author contributions

**Conceptualization:** Tao Xu.

**Data curation:** Lan Zhu, Shaomei Han.

**Formal analysis:** Tao Xu.

**Funding acquisition:** Lan Zhu.

**Investigation:** Tao Xu, Shaomei Han.

**Methodology:** Tao Xu.

**Project administration:** Tao Xu, Shaomei Han.

**Validation:** Tao Xu.

**Writing – original draft:** Tao Xu, Lei Zhang.

**Writing – review & editing:** Tao Xu, Zhiyi Li.
